# A pathway study of factors influencing quality of fertility life

**DOI:** 10.1186/s12889-024-18386-0

**Published:** 2024-04-15

**Authors:** Abulizi Maierhaba, Ming Jiang, Lihuan Zhi, Xueyu Wei, Lijuan He, Li Wang

**Affiliations:** 1 School of Public Health, Xinjiang Medical University, Urumqi, Xinjiang China; 2 The First Affiliated Hospital of Xinjiang Medical University, Urumqi, Xinjiang China

**Keywords:** Infertility, Fertility quality of life, Influencing factors, Pathway analysis

## Abstract

**Backgroud:**

To investigate the factors influencing fertility quality of life in infertile men, constructing a structural equation model of the factors influencing fertility quality of life in infertile men, and to provide suggested measures for improving fertility quality of life in infertile men.

**Methods:**

It is a Observational study. Infertile men (n = 250) attending a fertility centre in a hospital in Xinjiang, matched 1:2 men with no obvious male factor in the control group (n = 500).The Quality of Fertility Life Scale, the Social Support Scale, the Fertility Stress Scale and the Positive Attention Awareness Scale were used to conduct the survey. The model was constructed by applying the maximum likelihood estimation method in Mplus 8.3 software, to explore the factors influencing the quality of reproductive life of infertile men through path analyses. Differences between the case and control groups were statistically significant (*P* < 0.05) in terms of total fertility quality of life scores, core entry dimensions, affective responses, physical and mental relationships, selective treatment dimensions, and treatment tolerance.

**Results:**

Past medical history, history of exposure to hazardous environments, health insurance reimbursement, social support, fertility stress, and mindfulness are important factors affecting the quality of fertility life of infertile men.

**Conclusion:**

The quality of fertility life of infertile men is not optimistic. By improving the level of mindfulness, fertility stress, and social support, we propose appropriate measures to improve the quality of fertility life of infertile men. These measures can improve their confidence in clinical diagnosis and infertility treatment, enabling them to cope positively with these challenges.

## Introduction

According to the World Health Organisation, failure to conceive in a man and a woman who have lived together for more than one year, have a normal sexual life, and are not using contraception is called infertility [1]. People who meet this description are described as infertile. Rapid socio-economic development, rising living standards, and increasing social pressure, along with the increase of environmental pollution, drug abuse, poor dietary and lifestyle habits, and other influencing factors, have resulted in an increase in the number of infertility patients [2, 3]. The reproductive health of the infertile population is also regarded as a public health problem that needs worldwide attention [4]. In September 2020, the Expert Consensus on Male Reproduction-Related Genetic Tests issued by the Chinese Medical Association Men’s Branch showed that the incidence rate of male infertility in China is 10%∼15% [5].

Quality of life (QoL) is the most important topic to be addressed in infertility counselling [6], and its assessment and the identification of its influencing factors have become as important for treatment [7]. The results of a study on the QoL of Japanese men with infertility [8] showed that men with primary infertility had significantly lower scores in the emotional, physical, mental, and social domains, indicating a poorer QoL. Compared to them, Turkish men had significantly lower QoL, which may be related to the improvement of the treatment system for infertility and social subsidies in Japan [9]. In addition, the quality of fertility life of men with infertility in Middle Eastern countries such as Turkey and Iran is significantly lower, which is closely related to the traditional culture of each country [10]. Infertility brings a series of physical and psychological traumas to patients and families, which not only affects family happiness and harmony but also gradually affects social harmony [11].

At present, more studies exist on the quality of fertility life of infertile women than men. Additionally, the majority of these studies focus on examining the mechanism of the impact on the quality of life, with a dearth of literature addressing the pathways of the impact and positive thoughts. Many factors influence the quality of fertility life, and these factors are not mutually exclusive; instead, they interact with each other. They can have a direct effect on the quality of fertility life or an indirect effect through other factors. The purpose of this paper is to explore the influencing factors on the quality of reproductive life of male infertility patients, to construct a path model with the quality of reproductive life as the outcome variable, to explore the direct or indirect effects of exogenous potential variables on the outcome variable, and to clarify the path relationship between the variables, and one of the goals of this study is to determine whether the model proposed by the researchers is supported by the data in real conditions. To provide theoretical ideas for improving the quality of reproductive life of infertile men.

## Materials and methods

### Study population

This study was conducted using the case-control studies. Men attending the outpatient clinic of the Reproductive Fertility Centre of the First Affiliated Hospital of Xinjiang Medical University were recruited during the period of December 2020 to August 2023, with infertile men in the case group (n = 250) and men with no obvious male factor in the control group matched on a 1:2 basis (n = 500).

Inclusion criteria were men of reproductive age with normal sex life and not using contraception, regular menstruation of the female partner, no obvious abnormalities in gynaecological reproductive-related examinations [12]; abnormalities detected in routine semen examination (fifth edition of the World Health Organisation standards for semen analysis [13]); and providing informed consent and voluntary participation in the study.

Exclusion criteria were having a major disease [14]; having a proven psychological disorder, substance abuse or dependence history (including alcohol) [15]; and difficulty in understanding or an inability to complete the questionnaire.

### Survey instruments

#### General information questionnaire

Primary contents were age, ethnicity, place of residence, education level, weight, height, reproductive history, history of previous illnesses, monthly household income, occupation, history of smoking, history of alcohol consumption, exercise, sleep time, health insurance reimbursement, and history of exposure to hazardous environments.

#### Social support rating scale (SSRS)

The Social Support Rating Scale (SSRS) scale consists of 10 entries to calculate the total score of social support. The higher the score, the better the level of social support [16]. The Cronbach’s *a* coefficients of support rating scale was greater than 0.7, indicating good internal consistency, and the KMO value was greater than 0.7.

#### Massive attention and awareness scale(MAAS)

The MAAS is a scale developed by Ryan and Brown in 2003 [17]. It consists of 15 questions on a six-point Likert scale, with ‘1’ almost always and ‘6’ almost never, where the minimum score is 15 and the maximum score is 90. The higher the score, the higher the level of positive thinking.The Cronbach’s *a* coefficients of massive attention and awareness scale was greater than 0.7, indicating good internal consistency, and the KMO value was greater than 0.7.

#### Fertility pressure inventory (FPI)

The Fertility Problem Inventory (FPI) scale was used to measure the fertility-related stress of infertility patients, which was specially compiled to assess the fertility-related stress of infertility patients, with high reliability and validity of the total score and the subscales, which consisted of five dimensions with a total of 46 entries. The scale consists of 46 entries in five dimensions, including social pressure, sexual pressure, couple relationship, parental role demand, and rejection of childless lifestyle, with total scores ranging from 46 to 276, from ‘1 (totally disagree)’ to ‘6 (totally agree)’. The higher the score, the higher the level of fertility stress.The Cronbach’s a coefficient and KMO value of the Fertility Stress Scale are greater than 0.7, indicating that the questionnaire has good reliability and validity.

#### Fertility quality of life scale

The Fertility Quality of Life Scale consists of two modules (the core module and the selective treatment module) totalling 36 items [18]. The core module contains 24 items and four dimensions, with six items in each dimension; the selective treatment module contains 10 items, including the dimensions of therapeutic environment (six items) and tolerance (four items). Each entry is scored from 0 to 4, and the total score ranges from 0 to 100 by converting the scores of the total scale and subscales, and the standardised score is calculated by dividing the original score of the total scale × 25/number of entries in the total scale, with a higher score indicating a better QoL.The Cronbach’s a coefficient and KMO value of the Fertility Quality of Life Scale are greater than 0.7, indicating that the questionnaire has good reliability and validity.

### Statistical methods

Statistical analyses were performed in SPSS 25.0, and quantitative data were expressed as mean ± standard deviation. The t-test and one-way ANOVA were used for those conforming to normal distribution, and the rank-sum test was used for those parts not conforming to normal distribution to establish the multiple linear regression model. The maximum likelihood estimation method was applied to construct the model in Mplus 8.3 software to explore the fit of the actual data to the theoretical model as well as the direct and indirect effect paths and effect sizes. The Bootstrap procedure (5000 repeated extractions) was applied to test the existence of chained mediation effects, with 95% confidence intervals being the most important criterion for judgement, and the absence of 0 in the intervals means that the mediation effect is significant. The significance level was set at 0.05.

## Results

### Status of quality of reproductive life in the study population

The current status of the quality of reproductive life of the case group and the control group and the analysis of differences are shown in Table 1. The total score of the quality of reproductive life of the case group was significantly lower than that of the control group, of which the scores of the case group in the core entry dimension in terms of emotional response and physical and mental relationship were significantly lower than those of the control group, and the scores of the case group in terms of the selective treatment dimension were significantly lower than those of the control group. The case group scored significantly lower than the control group in treatment tolerance. (See Table 1)

**Table 1:** Analysis of the differences in quality of fertility life between the case and control groups

	Item	Case(n = 250)	Control(n = 500)	t	P
FertiQol	36	66.36 ± 11.81	68.35 ± 12.48	-2.093	**0.037**
Core FertiQol	24	70.68 ± 14.32	73.25 ± 15.40	-2.207	**0.028**
Physical condition	1	56.70 ± 17.57	57.95 ± 16.99	-0.939	0.348
Life condition	1	64.10 ± 20.13	63.30 ± 21.66	0.488	0.626
Physical and psychological health	6	70.67 ± 17.81	74.86 ± 18.91	-2.917	**0.004**
Emotional reaction	6	69.15 ± 19.10	73.73 ± 19.26	-3.075	**0.002**
Social relations	6	71.05 ± 15.14	70.37 ± 17.52	0.526	0.599
Marriage relations	6	71.85 ± 15.67	74.06 ± 16.46	-1.760	0.079
Treatment FertiQol	10	63.67 ± 14.54	66.25 ± 14.81	-2.264	**0.024**
Treatment environment	6	59.22 ± 14.17	59.64 ± 15.06	-0.371	0.710
Treatment endure	4	68.13 ± 20.33	72.86 ± 20.75	-2.967	**0.003**

### Univariate analysis of quality of reproductive life

The results of univariate linear regression showed that the score of quality of reproductive life in patients with a history of fertility was 6.139 units higher than that in patients without a history of fertility; the quality of reproductive life score for patients with a mean daily sleep time > 6 h was 6.743 units higher than that for patients with a mean sleep time ≤ 6 h. For every 1-unit increase in social support of patients in the case group, the corresponding quality of reproductive life increased on average by 0.621; for every 1-unit increase in the level of positive thinking of patients in the case group, their corresponding quality of reproductive life increased by 0.270; and for every 1-unit increase in the level of stress of fertility, the corresponding quality of reproductive life increased by 0.270. (See Table 2)

**Table 2:** Univariate analysis of the quality of reproductive life in the case group (n = 250)

Variant		N(%)	*x*±s	B	SE	95% CI of B	
						Lower limit	limit
**Age (years)**							
	≤ 32	150(60.00)	66.90 ± 10.85	Ref			
	> 32	100(40.00)	65.54 ± 13.13	-1.359	1.526	-4.364	1.645
**Nationality**							
	Han nationality	199(79.60)	66.22 ± 11.08	Ref			
	Minority	51(20.40)	66.87 ± 14.43	0.664	1.857	-2.993	4.322
**Fertility history**							
	No	230(92.00)	65.87 ± 11.82	Ref			
	Yes(≥1)	20(8.00)	72.01 ± 10.37	6.139	2.732	0.759	11.519^*^
**Body mass index**							
	≤ 18.5	4(1.60)	60.81 ± 3.33	Ref			
	18.5 ∼ 23.9	60(24.00)	64.16 ± 11.04	3.355	6.068	-8.597	15.307
	24 ∼ 27.9	113(45.20)	66.28 ± 12.20	5.475	5.978	-6.301	17.250
	≥ 28	73(29.20)	68.58 ± 11.82	7.775	6.034	-4.110	19.660
**Past medical history**							
	Yes	35(16.30)	60.56 ± 14.15	Ref			
	No	215(83.70)	67.30 ± 11.14	6.743	2.114	2.579	10.908^**^
**Diseases**							
	sexually transmitted disease	1(0.40)	71.88	5.540	11.854	-17.808	28.888
	Genitourinary infections	5(1.90)	67.40 ± 16.21	1.073	4.890	-8.559	10.705
	diabetes	2(0.80)	69.14 ± 7.92	2.806	8.401	-13.740	19.352
	Hypothyroidism or hyperthyroidism	2(0.80)	50.65 ± 32.59	-15.833	8.342	-32.264	0.598
	history of liver disease	13(5.20)	62.84 ± 10.59	-3.71	3.363	-10.334	2.914
	hypertensive	6(2.30)	60.04 ± 13.01	-6.494	4.519	-15.394	2.405
	Gastrointestinal diseases	3(1.20)	47.40 ± 19.14	-19.192	6.766	-32.518	-5.866^**^
	hormone abnormality	1(0.40)	74.74	8.416	11.847	-14.918	31.75
	cryptorchidism	2(0.80)	63.28 ± 4.42	-3.101	8.400	-19.646	13.445
	Orchitis, epididymitis	7(2.80)	60.04 ± 11.44	-6.494	4.519	-15.394	2.405
	No	215(83.70)	67.30 ± 11.14	Ref			
**Smoking**							
	Yes	106(42.40)	66.21 ± 12.13	Ref			
	No	144(57.60)	66.47 ± 11.62	0.256	1.515	-2.727	3.239
**Drinking**							
	Yes	85(34.00)	65.51 ± 13.36	Ref			
	No	165(66.00)	66.80 ± 10.95	1.291	1.578	-1.818	4.399
**Exercise**							
	Never or occasionally	122(48.80)	65.37 ± 12.21	Ref			
	1–2 times/week	89(35.60)	67.92 ± 11.49	2.546	1.648	-0.701	5.792
	3–4 times/week	26(10.40)	66.08 ± 11.89	0.703	2.554	-4.328	5.733
	≥ 5 times/week	13(5.20)	65.46 ± 10.07	0.092	3.450	-6.703	6.886
**Sleep**							
	≤ 6 h	40(16.00)	62.28 ± 13.17	Ref			
	>6 h	210(84.00)	67.13 ± 11.40	4.856	2.018	0.880	8.831^*^
**Marital status**							
	Unmarried	0(0.00)	0.00 ± 0.00				
	Married	249(99.60)	66.40 ± 11.82	10.148	11.842	-13.176	33.472
	Divorced	1(0.40)	56.25	Ref			
**Place of residence**							
	City	217(86.80)	66.21 ± 11.95	Ref			
	Rural	33(13.20)	67.32 ± 10.97	1.111	2.21	-3.242	5.464
**Education level**							
	Junior high school and below	20(8.00)	63.48 ± 15.51	Ref			
	High school/Secondary school	55(22.00)	64.39 ± 12.37	0.913	3.076	-5.147	6.972
	College/Undergraduate	163(65.20)	67.46 ± 11.15	3.983	2.791	-1.516	9.481
	Master’s degree and above	12(4.80)	65.21 ± 10.33	1.736	4.302	-6.738	10.210
**Occupation**							
	Institutions and establishments	61(24.40)	65.78 ± 11.89	0.708	2.108	-3.446	4.861
	Enterprise employee	63(25.20)	67.29 ± 9.96	2.216	2.091	-1.904	6.336
	Commercial service workers	13(5.20)	67.91 ± 10.23	2.838	3.576	-4.206	9.882
	Healthcare workers	6(2.40)	70.14 ± 6.27	5.068	5.015	-4.812	14.947
	Educator	6(2.40)	59.68 ± 18.23	-5.392	5.015	-15.272	4.487
	Transportation staff	12(4.80)	69.49 ± 9.55	4.417	3.697	-2.867	11.700
	Private sector employees	6(2.40)	69.36 ± 13.81	4.287	5.015	-5.593	14.166
	Self-employed	7(2.80)	59.82 ± 14.12	-5.25	4.677	-14.462	3.963
	Unemployed	1(0.40)	47.92	-17.154	11.831	-40.461	6.152
	Labour	4(1.60)	65.36 ± 6.03	0.293	6.052	-11.630	12.217
	Agriculture, fisheries and animal husbandry	8(3.20)	74.80 ± 7.48	9.734	4.406	1.055	18.413^*^
	Others	63(25.20)	65.07 ± 13.56	Ref			
**Monthly household income (yuan)**							
	< 4000	48(19.20)	64.43 ± 14.38	Ref			
	4000∼	103(41.20)	65.38 ± 10.45	0.951	2.057	-3.100	5.003
	7000∼	56(22.40)	68.14 ± 11.74	3.715	2.315	-0.845	8.275
	10,000∼	43(17.20)	68.54 ± 11.60	4.112	2.471	-0.756	8.980
**Exposure to harmful environments**							
	Yes	52(20.80)	62.39 ± 10.90	Ref			
	No	198(79.20)	67.40 ± 11.85	5.003	1.817	1.425	8.581^**^
**Type of medical insurance**							
	Yes	96(38.40)	69.05 ± 10.53	Ref			
	No	154(61.60)	64.68 ± 12.28	-4.373	1.514	-7.355	-1.391^**^
**Social support**				0.621	0.105	0.414	0.829^***^
**Mindfulness**				0.270	0.058	0.157	0.383^***^
**Fertility pressures**				-0.108	0.028	-0.163	-0.052^***^

Unitary linear regression was used to analyse the factors influencing the emotional response dimension, physical and mental relationship dimension, and treatment tolerance dimension of the quality of reproductive life of the men in the case group, and the results showed the following:

In the emotional response dimension, no history of disease and having a history of childbearing were the factors influencing the emotional response; in the physical and mental relationship dimension, no history of disease and having a history of childbearing were the factors influencing the physical and mental relationship; and in the treatment tolerance dimension, treatment tolerance was higher in men with reproductive history.

In the affective response dimension, the mean increase in affective response was 0.284 units for every 1-unit increase in positive thoughts; the mean decrease in affective response was 0.095 units for every 1-unit increase in fertility stress; and in the mind-body relationship dimension, the mean increase in mind-body relationship was 0.343 units for every 1-unit increase in positive thoughts; and the mean decrease in mind-body relationship was 0.151 units for every 1-unit increase in fertility stress.

The mind-body relationship was 6.677 units higher in patients with an average sleep duration of > 6 h/day than in patients with an average sleep duration of ≤ 6 h/day; in the dimension of treatment tolerance, for each increase of 1-unit in positive thoughts, the mean increase in treatment tolerance was 0.370 units. For every 1-unit increase in social support, emotional response, physical and mental relationships, and treatment tolerance increased by a mean of 0.450, 0.565, and 0.534 units, respectively.

In the dimension of emotional response, monthly family income of 10,000 yuan and above positively affected emotional response; in the dimension of physical and mental relationship, monthly family income of 10,000 yuan and above, and no history of exposure to harmful environments were factors affecting physical and mental relationship, and students’ physical and mental relationship was 20.685 units lower than the physical and mental relationship of patients with other occupations.

In the dimension of treatment tolerability, the self-employed, those engaged in agriculture, fishery, and animal husbandry, monthly household income of 7000 ∼ 10,000 yuan, and no history of hazardous environmental exposure were the influencing factors of treatment tolerance. Patients without health insurance reimbursement scored lower than patients with health insurance reimbursement in the dimensions of emotional response, physical and mental relationship, and treatment tolerance.

### Multifactorial analysis of quality of fertility life

Based on unitary linear regression, statistically significant variables were included in the multiple linear regression model, with quality of reproductive life as the dependent variable, which showed that the adjusted R^2^ was 0.249, the independent variable explained 24.9% of the dependent variable. No history of illness, positive thoughts, social support, and no history of harmful environmental exposure, fertility stress, and no health insurance reimbursement were the influencing factors of QoL in childbearing. (See Table 3)

**Table 3:** Multiple linear regression analysis of quality of fertility life in the case group

Variant	Unstandardized coefficient		β	t	P	95.0% CI of B	
	B	SE				Lower limit	Limit
(Constant)	36.489	9.267		3.938	0.000	18.234	54.744
Fertility history	4.754	2.477	0.109	1.919	0.056	− 0.126	9.634
Past medical history	4.807	1.935	0.141	2.484	**0.014**	0.995	8.620
Sleep	3.084	1.796	0.096	1.717	0.087	− 0.454	6.622
Mindfulness	0.164	0.056	0.173	2.914	**0.004**	0.053	0.275
Fertility pressure	− 0.054	0.027	-0.119	-2.015	**0.045**	− 0.107	− 0.001
Occupation: farming, fishing and herding	6.659	3.766	0.099	1.768	0.078	− 0.759	14.076
Social support	0.409	0.102	0.230	3.990	**< 0.001**	0.207	0.610
Exposure to harmful environment	3.977	1.615	0.137	2.463	**0.014**	0.797	7.158
Medical insurance reimbursement	− 3.152	1.370	-0.130	-2.302	**0.022**	− 5.850	− 0.455

Statistically significant variables were included in the multiple linear regression model with affective response as the dependent variable, and the results showed that the adjusted R^2^ was 0.117, which means that the independent variable explained 11.7% of the dependent variable. History of childbearing, no history of illness, positive thoughts, and no health insurance reimbursement were the influencing factors of affective response.

Statistically significant variables were included in the multiple linear regression model, with the mind-body relationship as the dependent variable, and the results showed that the adjusted R^2^ was 0.141, which means that the independent variable explained 14.1% of the dependent variable. No history of illness, positive thoughts, and no health insurance reimbursement were the factors affecting the mind-body relationship.

Statistically significant variables were included in the multiple linear regression model, with treatment tolerance as the dependent variable, and the results showed that the adjusted R^2^ was 0.119, which means that the independent variable explained 11.9% of the dependent variable. Positive thoughts, not working in agriculture, fishery, or pastoral labour, no history of harmful environmental exposure, and no health insurance reimbursement were the factors affecting treatment tolerance.

### Path analysis of reproductive quality of life

Path analysis is an extension of multivariate linear regression analysis, which does not require the variables to be independent of each other, and it can accommodate the causal structure of multiple links. Consequently, it can represent these causal relationships very clearly through path diagrams, according to which deeper analyses can be conducted, and it is suitable for analysing multivariate dependence problems that contain indirect influence relationships [19].

**Fig. 1:**
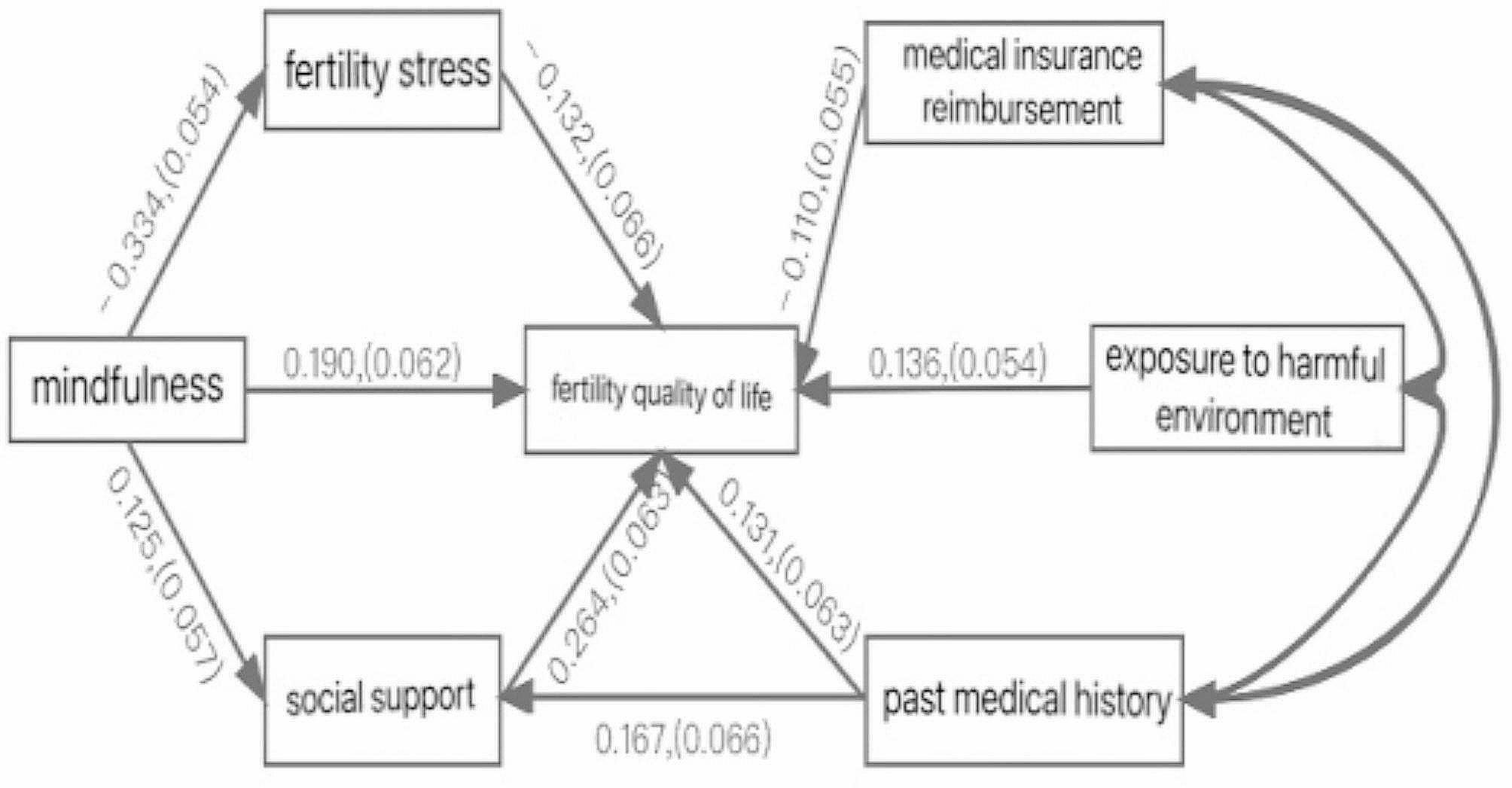
Path analysis of reproductive quality of life

The main reference fit indicators in structural equation modelling were CFI > 0.9; TLI > 0.9; RMSEA < 0.06; SRMR < 0.08; 𝟀2/df is the composite fit indicator, 𝟀2/df < 3 is the best fit, and 𝟀2/df < 5 is a fit that is moderate (*N* ≥ 250). The established hypothetical model was tested for the goodness-of-fit of the model and model correction several times, and the fitting results of the final impact path theoretical model and the actual data showed that they all met the required fit criteria. The specific indexes were: 𝟀2/df = 1.56, CFI = 0.968, TLI = 0.920, RMSEA = 0.047, SRMR = 0.045, and the model fit is good. The path model is shown in Fig. 1.

The test results of mediating effect of path analysis of infertile men’s reproductive QoL are shown in Table 4. The standardised path coefficient from mindfulness to fertility stress is -0.334, from mindfulness to social support is 0.125, from mindfulness to fertility quality of life is 0.190. Similarly, all other standardised path coefficients can be seen in Fig. 1.

**Table 4:** Direct and indirect effects of path diagrams

Pathway	Standardized	P	95%CI
Past medical history$$ $$\rightarrow$$ $$FertiQol			
Overall effect	0.175	0.006	0.045∼0.296
Total indirect effect	0.044	0.034	0.011∼0.095
FertiQol$$ $$\leftarrow$$ $$Social support$$ $$\leftarrow$$ $$Past medical history	0.044	0.034	0.011∼0.095
FertiQol$$ $$\leftarrow$$ $$Past medical history	0.131	0.028	0.003∼0.253
Mindfulness$$ $$\rightarrow$$ $$FertiQol			
Overall effect	0.267	< 0.001	0.151∼0.373
Total indirect effect	0.077	0.009	0.019∼0.138
FertiQol$$ $$\leftarrow$$ $$Fertility pressure$$ $$\leftarrow$$ $$Mindfulness	0.044	0.042	0.001∼0.087
FertiQol$$ $$\leftarrow$$ $$Social support$$ $$\leftarrow$$ $$Mindfulness	0.033	0.061	0.005∼0.075
FertiQol$$ $$\leftarrow$$ $$Mindfulness	0.190	0.002	0.065∼0.306

Path 1: Disease history can directly affect the reproductive QoL and also indirectly affect the reproductive QoL through social support, the value of direct path effect is 0.131, which accounted for 74.86% of the total effect size. The 95% confidence interval for the simple mediation effect of disease history→social support→quality of reproductive life was (0.011, 0.095), and the interval did not contain 0, indicating that the mediation effect of this indirect pathway was significant, and that disease history positively predicted quality of reproductive life through social support. The total indirect effect value was 0.044, accounting for 25.14% of the total effect size.

Path 2: mindfulness can directly affect the quality of reproductive life, but also indirectly affect the quality of reproductive life through the two paths of reproductive stress and social support, the direct path effect value is 0.190, accounting for 71.16% of the total effect size. The 95% confidence interval for the simple mediation effect of Positive Thoughts→Fertility Stress→Quality of Reproductive Life is (0.001, 0.087), and the interval does not contain 0, indicating that the mediation effect of this indirect path is significant. The value of the indirect path effect is 0.044, indicating that its effect on the quality of reproductive life through the mediation of fertility stress increases by 0.044, accounting for 16.48% of the total effect; the 95% confidence interval for the simple mediation effect of Positive Thoughts→Social Support→Quality of Reproductive Life is (0.005, 0.075), with an interval that does not contain 0, indicating that the mediation effect of this indirect path is significant. The indirect path effect value is 0.033, indicating that its effect on the quality of reproductive life through the mediation of social support increased by 0.033, accounting for 12.36% of the total effect; positive thoughts positively predicted the quality of reproductive life through fertility stress and social support.

## Discussion and conclusion

Under the special cultural background of China, it is believed that the transmission of a family is a major event in life, and infertility brings great mental trauma to the patients themselves as well as great pain to both families [14]. Infertility is a reproductive disease that involves the privacy of the patient, and the treatment process is often a combination of hope and disappointment, during which the patient’s psychological fluctuations are large, resulting in psychological stress and thus reducing the quality of reproductive life [20].

We explored the factors and pathway analysis affecting the quality of reproductive life of infertile men based on the health ecology model. The core entry dimensions between our case groups and control group differed significantly in emotional response and physical and mental relationships. The emotional response score of men in the case group was lower than the study of 304 infertile men by Cai and Dong [21]. The score of physical and mental relations in the case group was lower than the control group, and likewise lower than the results of the survey conducted by Cusatis et al. [22] in 2018 in the US.

Infertility itself and its treatment process will cause great damage to the body and mind, affecting their QoL [23]. The score of treatment environment in the selective treatment dimension was lower than 60. Healthcare professionals should try to create a harmonious and warm medical environment for male infertility patients [24]. A good environment for sperm retrieval examination and treatment is especially important for male infertility patients to face the disease more positively. Infertile men face long-term infertility treatment and suffer from great social and psychological pressure, and the length of treatment and repeated treatments also significantly affect the QoL of the patients’ fertility.

Long-term repeated treatments may lead to mental health problems and even cause a decrease in the conception rate [25, 26]. The difference in quality of reproductive life between the case and control groups was statistically significant. Under the traditional Chinese concept, not having children is not considered as a complete family, and the disease itself brings multiple pressures on the patients such as a long duration of the disease and a large financial burden, which greatly reduce the quality of reproductive life of male infertility patients [23].

By developing structural equation modelling, we showed that infertile men’s past disease history not only directly affects their reproductive QoL, but disease history also affects reproductive QoL by influencing social support, which in this paper indicates that men with no history of disease receive more support in their social network relationships and thus improve their reproductive QoL. Positive thoughts directly affect the quality of reproductive life and can also indirectly affect the quality of reproductive life by affecting fertility stress. The higher scores of parental role demands in fertility stress among the study participants may be related to the traditional concept in China that men, as the breadwinners of the family, reproducing offspring is a major role expectation, while infertile men experience fertility-related stress due to fertility problems [21].

Although some infertility can be improved and cured through later treatment, due to social prejudice, infertile men avoid their condition due to inferiority complex, rarely discuss their condition with doctors and family members, and actively accept appropriate treatment, resulting in physical and mental pain [27]. Positive thoughts → fertility stress → quality of reproductive life, indicating that the indirect effect of positive thoughts was weakened after fertility stress mediation. Consistent with the results of a previous study [28], patients with high levels of positive thoughts can better regulate their emotions and alleviate the stress of childbirth, which helps to eliminate intense stress and improve QoL. Positive thoughts can indirectly affect the quality of reproductive life through social support, and studies have shown [29] that improving the quality of reproductive life of infertile men requires a high level of support from spouses and family members. Therefore, the attention to the patient can be improved by establishing certain social support networks so that the patient can increase his self-confidence to cope with the disease treatment process. Medical personnel can conduct interventions from the perspective of enhancing social support, which can improve the QoL of patients and enhance the recognition of self [30].

This study has the following limitations: (1) This study only focused on infertile men who came to the hospital to seek treatment for infertility making the findings somewhat biased; (2) it was limited to infertile men in the Reproductive Centre of the First Affiliated Hospital of Xinjiang Medical University; (3) it is a cross-sectional study; and (4) it only examined the quality of reproductive life of infertile men but did not investigate their spouses at the same time, ignoring the mutual influence and differences between spouses and infertile men in the quality of reproductive life.

Future research could include the following: (1) Building a WeChat platform to incorporate more cases, (2) promoting the use of samples at all levels of hospitals and in all communities, (3) including a longitudinal intervention study, and (4) including infertile men’s spouses.

## Data Availability

The datasets generated and/or analysed during the current study are not publicly available due considere private to the patients. but are available from the corresponding author on reasonable request.
